# Visual distractions for pediatric patients in a dental environment using eye-tracking technology: a cross-sectional study

**DOI:** 10.1007/s00784-026-07019-y

**Published:** 2026-08-27

**Authors:** Alhowra A. Alshaikh, Ghalia Y. Bhadila, Osama M. Felemban, Heba M. Elkhodary, Amani A. Al Tuwirqi

**Affiliations:** 1 Dental Department, Jubail Health Network, Ministry of Health, Al Jubail, 31951 Saudi Arabia; 2 Pediatric Dentistry Department, Faculty of Dentistry, King Abdulaziz University, Jeddah, 21589 Saudi Arabia; 3 Department of Pedodontics and Oral Health, Faculty of Dental Medicine for Girls, Al Azhar University, Cairo, 11651 Egypt

**Keywords:** Children, Dental anxiety, Eye-tracking, Gaze behavior, Distraction

## Abstract

**Objective:**

To evaluate visual attention patterns in anxious versus non-anxious children toward various environmental distractions in a dental clinic using eye-tracking technology and assess the impact of visual distractors on gaze behavior.

**Materials and methods:**

This cross-sectional study, including 121 children 6–12 years of age. Anxiety levels were determined using the Children’s Fear Survey Schedule–Dental Subscale. Standardized operatory images, with and without distractors, were presented while eye-tracking metrics were recorded. Heatmaps were generated and group comparisons were performed using non-parametric tests.

**Results:**

Nearly one-half (49.6%) of participants were classified as anxious who, in the absence of distractors, exhibited faster entry times and longer dwell times on dental instruments and handpieces than their non-anxious counterparts (*p* < 0.001). Introduction of distractors significantly redirected gaze toward child-friendly elements, altered fixation sequences, and reduced revisits to clinical tools. Children in both groups consistently focused on clinicians’ eyes, with decorated masks attracting further attention.

**Conclusion:**

Anxious children demonstrated distinct gaze patterns compared with their non-anxious peers, exhibiting faster orienting and altered engagement with dental instruments. Introduction of visual distractors significantly redirected attention toward child-friendly elements and away from anxiety-provoking stimuli, emphasizing the influence of anxiety on visual focus and the effectiveness of environmental modifications in improving the pediatric dental experience.

**Clinical relevance:**

This study contributes to the design of anxiety-reducing pediatric dental environments by identifying elements that attract the visual attention of children. The presence of appropriate distractors within the pediatric dental clinic may help redirect children’s gaze away from clinical tools and instruments. These findings can inform the design and layout of pediatric dental clinics to enhance comfort and patient cooperation, ultimately improving the overall quality of pediatric dental care.

## Introduction

Dental anxiety (DA) describes patients’ reactions to the stress associated with dental procedures, especially when the stimulus is ambiguous, unidentified, or not immediately present [1]. Children and adolescents who fear their dentist may exhibit negative behaviors during examinations and procedures, ranging from small-scale restlessness to outbursts [1]. Children’s anxiety levels affect treatment outcomes, and their behaviors reflect their psychological states. Therefore, pediatric dentists are responsible for implementing suitable behavioral management techniques based on in-depth assessments and counseling during dental visits. Negative behavior in dental settings may result from the absence of previous visits, poor oral hygiene, or earlier painful procedures, whereas regular visits and a friendly dental team help reduce DA [2]. DA assessment, therefore, is essential for diagnosis and behavioral management; therefore, several validated scales have been developed to evaluate anxiety in children. However, these tools are typically used when clinically or research-wise indicated rather than routinely before every dental visit, as unnecessary focus on fear may influence children’s emotional responses. The Children’s Fear Survey Schedule–Dental Subscale (CFSS-DS) is a reliable tool validated in the Arabic language [3]. The CFSS-DS has demonstrated high reliability and validity across cultures, including Arabic-speaking populations, and effectively differentiates fearful from non-fearful children [3]. Earlier studies have investigated various factors influencing DA, such as parental anxiety, prior medical experiences, individual temperament, and the characteristics of the dental environment, underscoring the complex nature of dental fear in children [4–6]. Thus, the etiology of DA in children is multifactorial and extends beyond previous dental experiences. Factors such as limited cognitive maturity or impaired understanding of dental procedures may also contribute to the development of DA. DA tends to decrease as children grow older, likely because repeated exposure to dental visits increases familiarity and reduces fear. In the same study, younger children (7–8 years old) showed higher anxiety levels than older children (11–12 years old), highlighting how DA can lessen with age and developmental maturity [7]. Moreover, repeated dental visits helped reduce DA over time [8]. Injections, drilling, and the sight of dental instruments are among the most commonly reported sources of fear. These fears tend to intensify if the child has had previous unpleasant or painful dental experience [9, 10].

Distraction is an effective behavioral guidance technique for reducing DA in children by diverting attention from unpleasant procedures [11]. Audiovisual strategies, including virtual reality, minimize anxiety by immersing patients in interactive environments [12]. A supportive treatment environment, such as color, lighting, sound, and family presence, also reduces anxiety and enhances comfort. Family presence may variably influence children’s responses during dental treatment, as supportive parents can enhance cooperation and reduce anxiety, whereas anxious or distressed parents may increase fear; therefore, understanding the effect of parental presence or absence is essential to optimize cooperation, communication, and treatment experience [13, 14]. Warm and cool colors, such as yellow and blue, can promote calmness, while harsh colors, such as black and red, may increase discomfort [15].

In pediatric dentistry research, eye tracking is valuable for assessing visual attention and emotional responses when self-reporting is unreliable [16, 17]. Metrics such as dwell time, fixation sequence, and revisits reveal the operatory elements that attract attention [18]. Children tend to fixate longer on instruments when colorful distractors or screens redirect their attention [12, 16]. These non-invasive measures offer a quantifiable evaluation of interventions, contributing to a better design of pediatric dental environments [16, 17]. However, no studies have specifically evaluated what anxious children notice the most during a pediatric dental procedure, representing a clear research gap.

We relied on the “eye-mind” hypothesis, a foundational concept in eye-tracking research which posits that there is a strong link between where a person is looking (gaze fixation) and what they are cognitively processing as areas of high interest (visual attention) [17]. This methodology is well-established in dental research for assessing attention to caries [17], orthodontic aesthetics [18], and cleft lip repair [19].

The present study aimed to evaluate the visual attention patterns of children 6–12 years of age in a dental operatory using eye-tracking technology by comparing anxious and non-anxious children, and to assess the effect of visual distractors such as cartoons, screens, and wall stickers. Identifying the visual elements that attract anxious children can guide the design of calming environments and improve cooperation and quality of care. We hypothesized that there would be a difference between anxious and non-anxious children regarding what they notice the most in a dental operatory setting, and that there would be a positive effect of introducing distractors to the dental clinic on children’s gaze patterns assessed using eye-tracking technology.

## Materials and methods

### Study design, setting, and ethics

This cross-sectional study was conducted with children aged 6–12 years attending pediatric dental clinics at King Abdulaziz University Dental Hospital (KAUFD), Jeddah, Saudi Arabia, between February and October 2024. Ethics approval was obtained from the Research Ethics Committees at KAUFD (REC# 275-12-23; approval date: February 18, 2024). Informed written consent was obtained from parents or legal guardians, and age-appropriate verbal assent was obtained from the children before participation. This study was conducted and reported in accordance with the Strengthening the Reporting of Observational Studies in Epidemiology (STROBE) for cross-sectional studies.

### Participants, eligibility, and sample size

Participants were recruited using convenience sampling. Healthy children aged 6 to 12 years attending the pediatric dental clinics for dental visits between 20 February 2024 and 22 October 2024 were approached and screened for eligibility based on age and willingness to participate. A total of 131 eligible patients were approached; only 10 patients refused to participate. Therefore, the final sample included 121 participants.

Children were excluded from participating in the study if they had known visual, hearing, or developmental disorders; any related medical conditions or syndromes; if they or their parents/legal guardians declined to provide assent or informed consent; or if they failed to complete the eye-tracker calibration process. Eligible participants who provided assent and whose parents gave informed consent were administered the Arabic version of the CFSS-DS to determine their level of anxiety.

Sample size estimation was based on previous pediatric dental eye-tracking data [16], assuming a mean difference of 0.5 ± 0.67 s between groups in eye-tracking parameters would be significant, and 59 participants per group were required to achieve 95% power at a significance level of 0.05 using independent sample means testing. Data were collected using convenience sampling. Parents were approached in the pediatric waiting area, informed of the study objectives, and invited to participate. Consent and assent were obtained before enrollment.

### Anxiety assessment and group allocation

To determine anxiety levels, children were assessed using the validated Arabic version of the CFSS-DS, which contains 15 items rated on a five-point Likert scale, with scores ranging from 15 to 75. Scores < 38 indicate non-anxiety, whereas scores ≥ 38 indicate anxiety [11]. Children were categorized into 2 groups based on dental anxiety level: anxious and non-anxious.

### Pilot study

Before commencing data collection, a pilot study was conducted with 10% of the intended sample to assess questionnaire reliability, confirm the timing of image presentation, obtain feedback from children, and verify the accuracy of eye-tracking data export. This phase confirmed the feasibility of the study protocol and refined the logistical aspects before data collection.

### Experimental stimuli and image preparation

**Fig. 1:**
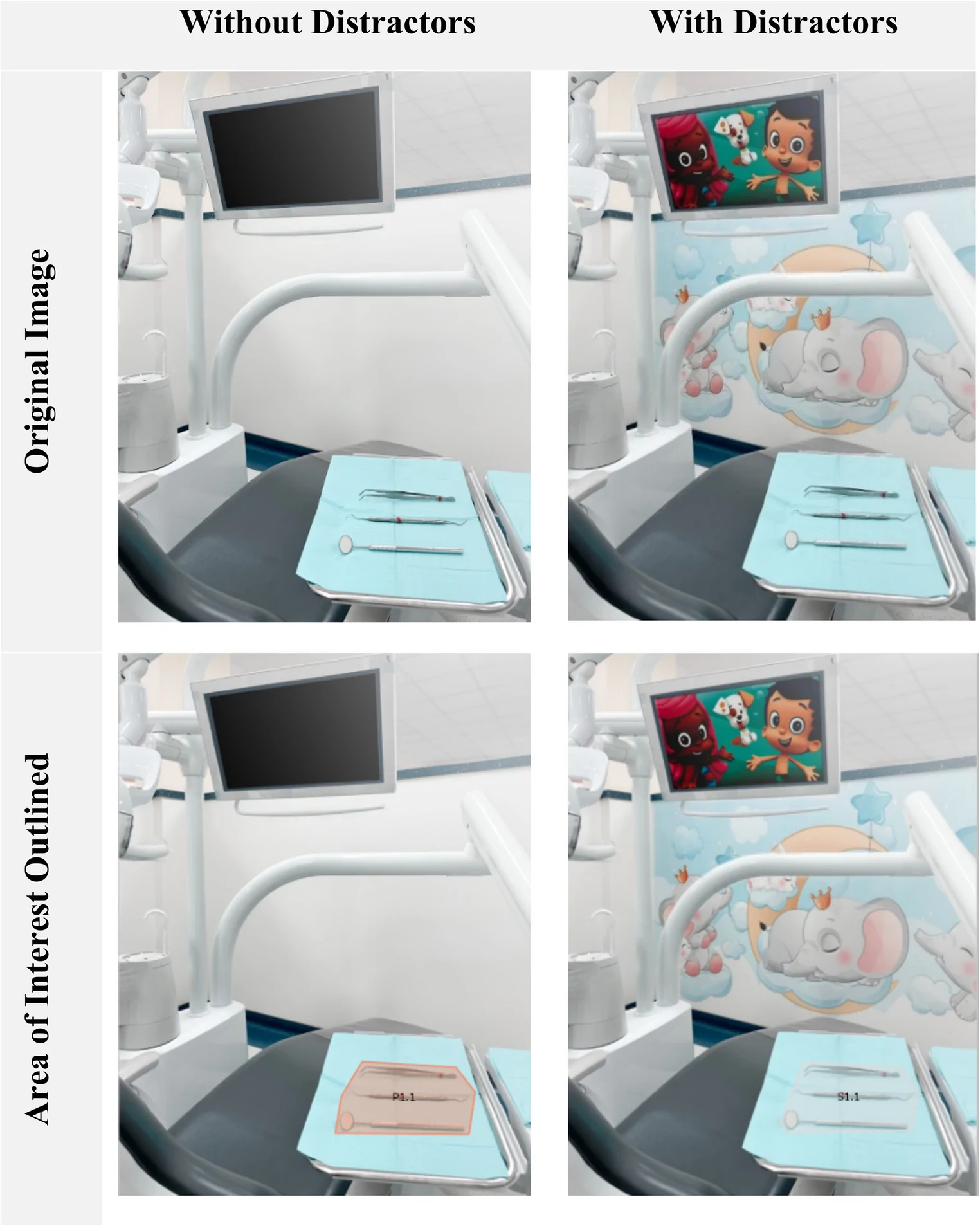
Close-up view of dental instruments: with and without wall and screen distractors, including area of interest (Instruments) delineations

**Fig. 2:**
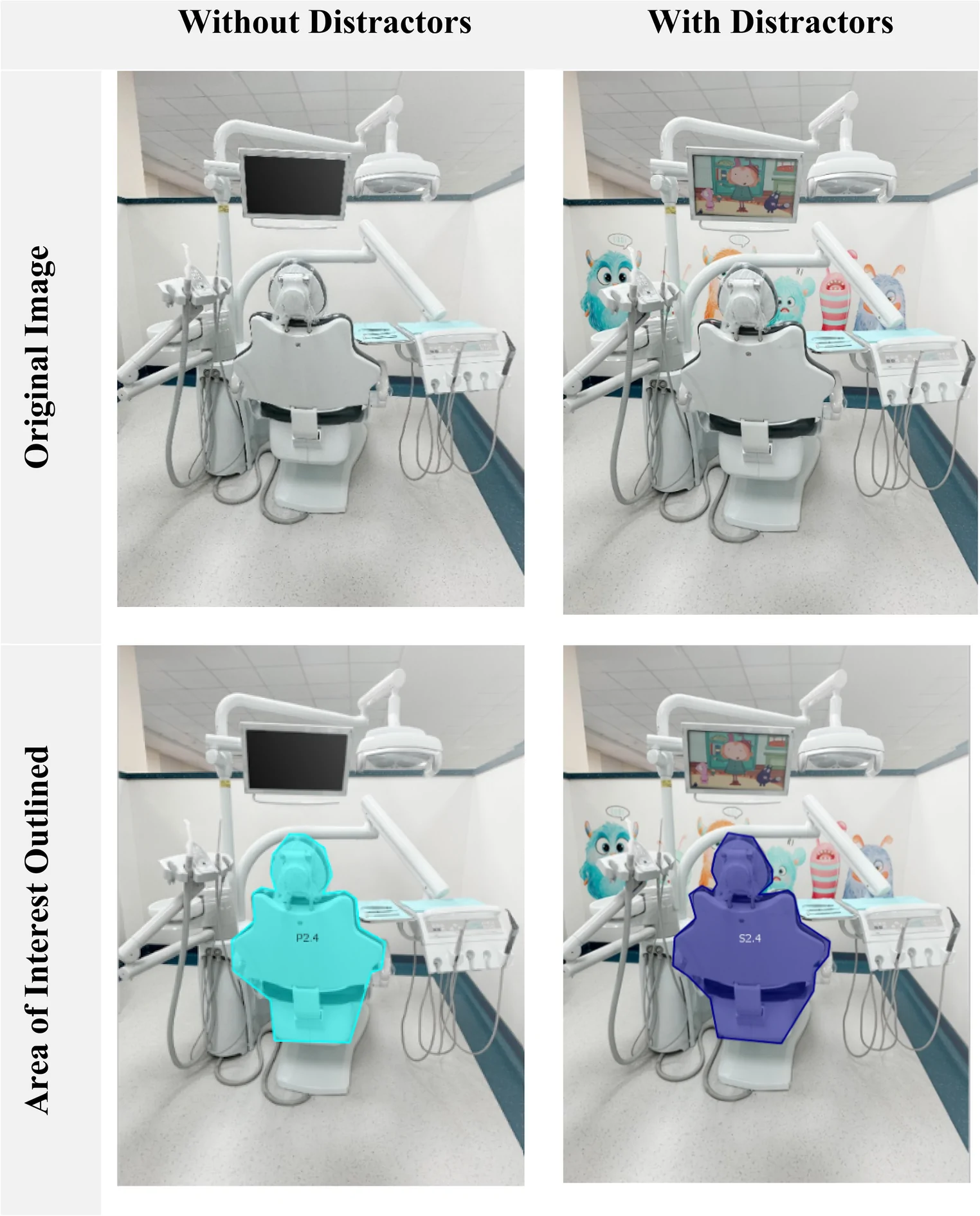
Entrance perspective of the clinic: with and without wall and screen distractors, including area of interest outlines

**Fig. 3:**
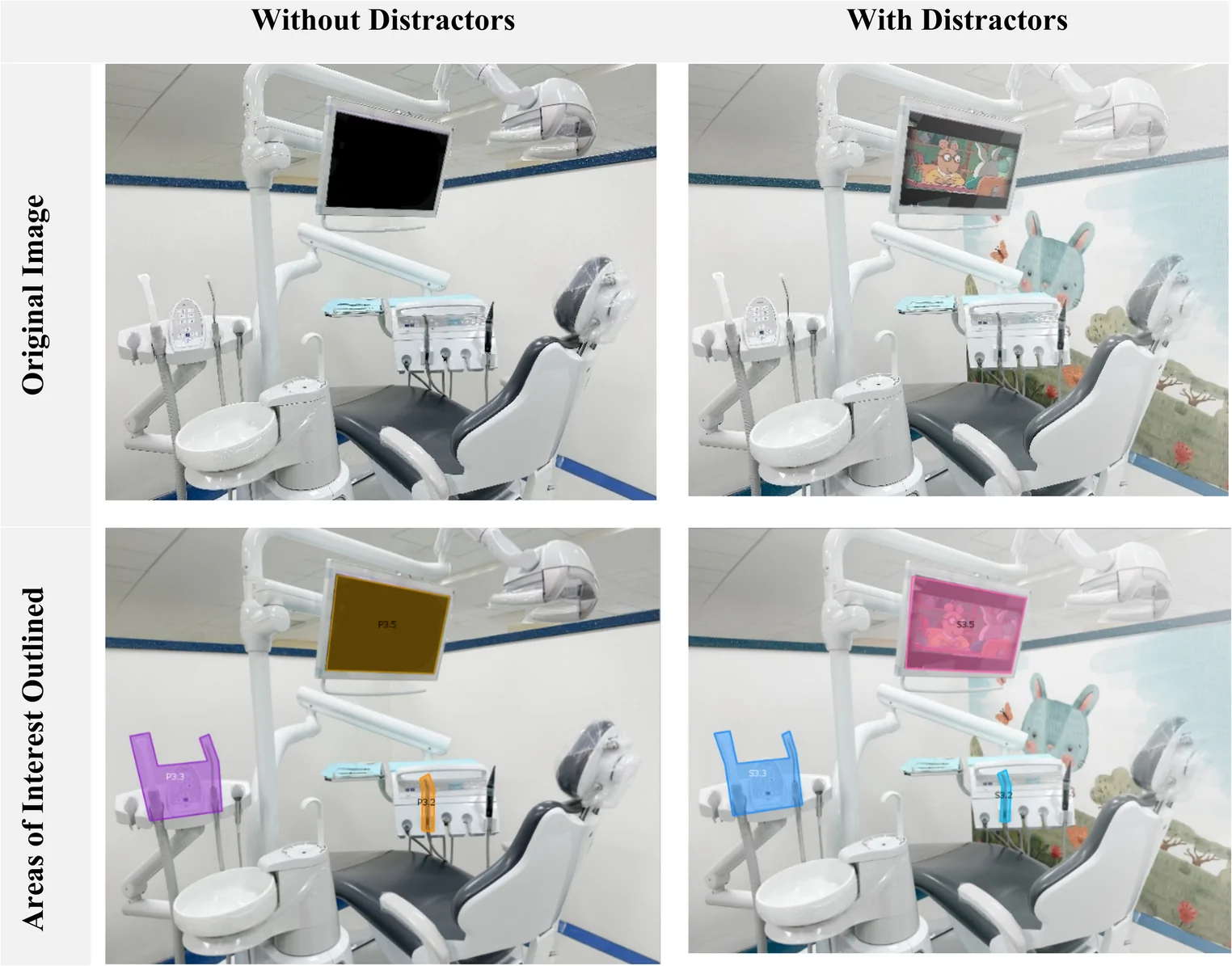
Side view of the dental clinic: with and without wall and screen distractors, including area of interest mappings

**Fig. 4:**
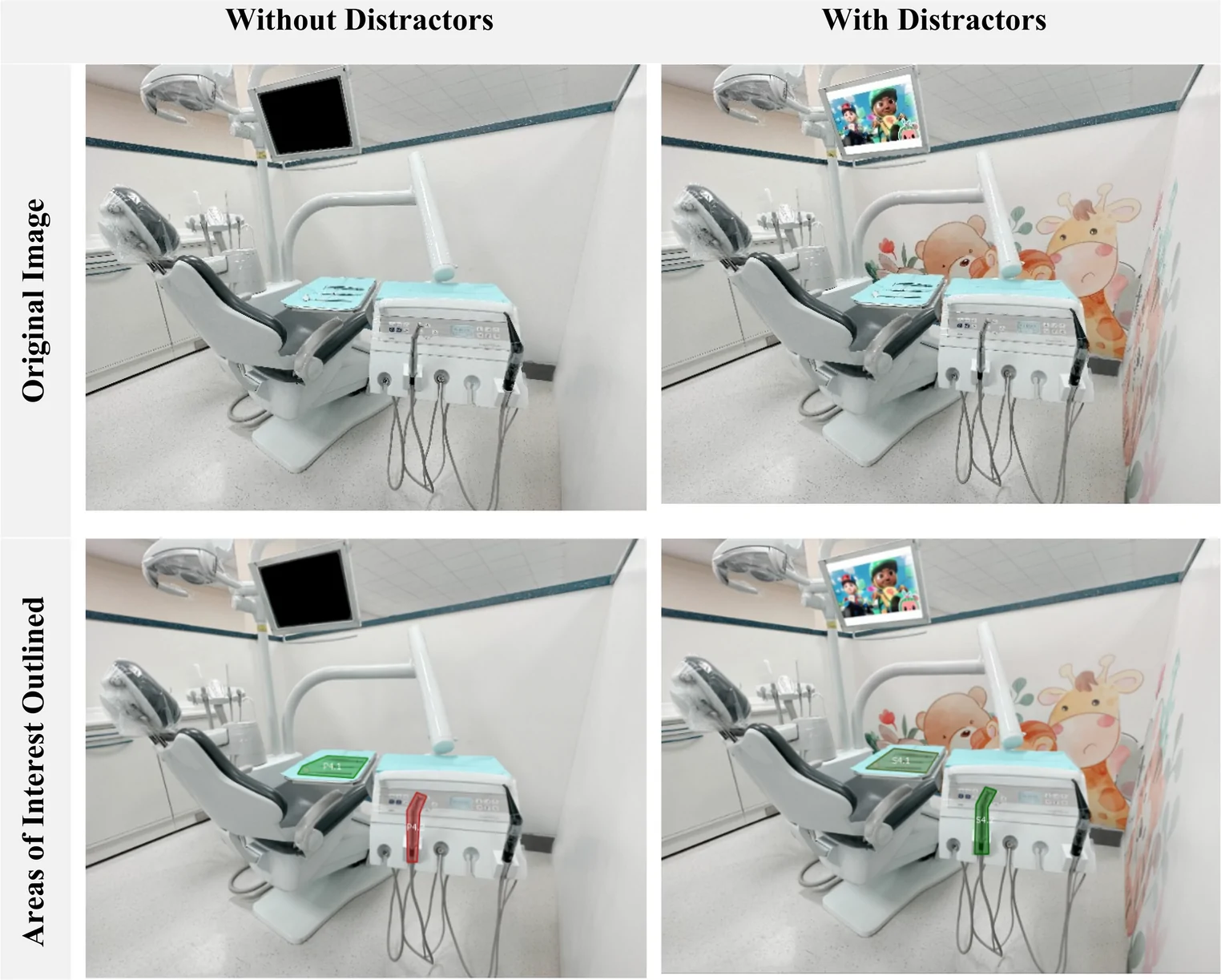
Alternative perspective of the dental clinic: with and without wall and screen distractors, including area of interest outlines

**Fig. 5:**
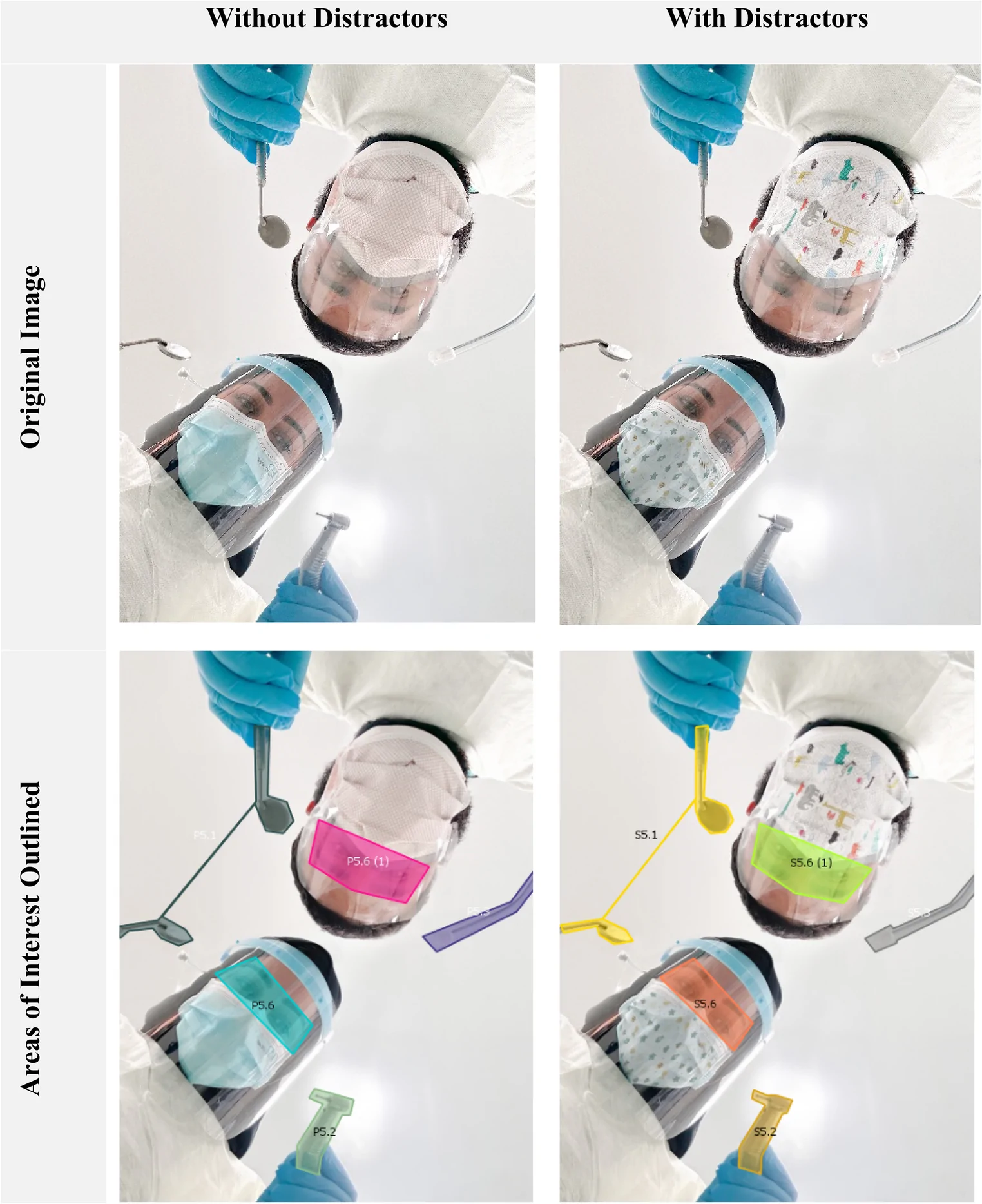
Patient’s perspective in the dental chair under examination: with and without face mask distractors, including area of interest outlines

The eye-tracking tool was used to objectively measure and analyze participants’ gaze behavior while viewing standardized images of a dental operatory under two conditions: a standard clinical setup, and an environment enhanced with visual distractors (e.g., colorful designs and child-friendly elements). A standardized set of pediatric dental operatory images was captured from various angles. Each image also had a digitally modified version that incorporated visual distractors (e.g., colourful wall paintings and cartoon images) to evaluate gaze differences. The images included exploratory dental instruments, a light dental chair, a handpiece and suction, a dental unit with a high-speed handpiece and suction, a dental chair with a cupboard, and two dental operators wearing masks and face shields. All images were captured at approximately 120 cm to simulate the child’s perspective, either from the dental chair or from the operatory doorway [16]. The areas of interest (AOIs) were mapped for each photograph to identify specific elements within the dental operatory as follows: bracket table (Appendix A, Fig. 1); dental chair (Appendix A, Fig. 2); suction, handpieces, and screens (Appendix A, Fig. 3); instruments and handpieces (Appendix A, Fig. 4); and the operators’ eyes, held instruments, handpieces, and suction (Appendix A, Fig. 5). This mapping enabled the evaluation of gaze patterns within these regions, including metrics such as entry time, dwell time, fixation sequence, and revisits.

### Eye-tracking procedure

To assess participant visual attention patterns, eye movements were recorded using a remote eye-tracker (RED-m, Sensomotoric Instruments (SMI), Teltow, Germany) mounted at the base of a 15.6-inch laptop screen (Latitude E6530, Dell Corporation, Round Rock, TX, USA) with a screen resolution of 1600 × 900 pixels. The system tracked both eyes and was compatible with most prescription glasses and contact lenses. Data collection was performed using Experiment Center 3.7, and the analysis was performed using BeGaze Software (SMI, Germany). The data were automatically imported from the experimental platform into the analysis software for processing. The experiment was performed in a quiet, dimly lit room. The participants were seated approximately 60 cm away from a wall-mounted laptop and instructed to remain still and focus on the images. Calibration was performed using a five-point system and repeated as needed until a precision of ≤ 0.9° was achieved. All participants were asked to observe passively, and no interaction was required. Operatory images were presented in random order for each subject, and unrelated distractor images, such as landscapes, were interspersed to minimize habituation. Each image was displayed for 5 s [16], and the total duration of the experiment, including calibration, was approximately 65 s per participant.

### Outcome measures and eye-tracking metrics

Heatmaps illustrating fixation concentration and distribution were automatically generated using eye-tracking software. These qualitative outputs were visually assessed to identify the operatory features that consistently attracted attention, with consideration of the presence or absence of visual distractors. Quantitative data were extracted for predefined areas of interest, including entry time, dwell time, sequence, and revisits, as defined by established eye-tracking methodology. Entry time refers to the interval between image presentation and the first fixation on an area of interest, whereas the dwell time represents the cumulative time devoted to viewing the area, including all fixations and saccades. The sequence reflected the chronological order of the first fixations across the AOIs, and revisits referred to the number of times participants returned to a previously fixated area. These metrics were summarized and compared between the anxious and non-anxious groups, as well as between images with and without visual distractors. Each image was visible for 5 s, and each participant took approximately 65 s to complete the entire experiment (including calibration and distractor picture presentation time). The experiment ended for each participant when all photographs were deemed complete on the screen.

### Statistical analysis

Continuous variables are expressed as mean ± standard deviation (SD) or median with interquartile range (IQR), while categorical variables are reported as frequency and percentage. Nonparametric tests were used because the data were not normally distributed. The Mann–Whitney U test was used for between-group comparisons, the Wilcoxon signed-rank test for paired data, and the Friedman test for repeated-measures data. Associations between categorical variables were evaluated using the chi-squared test. Differences with *p* ≤ 0.05 were statistically significant. Statistical analyses were performed using SPSS version 24.0 (IBM Corp., Armonk, NY, USA) for Windows. Heatmaps of the fixation concentration over time were automatically generated using eye-tracking software and exported to each participant. The analysis was descriptive and observational, complementing the quantitative gaze data.

## Results

A total of 121 children were included in the final sample, with 60 classified as anxious and 61 as non-anxious. The mean age was 8.7 ± 1.8 years, with nearly equal distribution between 6 and 8 (50.4%) and 9 to 12 (49.6%) years’ age groups. Females represented 57.0% of the sample, and 90.9% had previous dental visits. Younger children (6–8 years of age) were more likely to be anxious than their older counterparts, and anxiety was more common among females. Although most participants in both groups had a previous dental experience, a greater proportion of anxious children reported no previous dental visits.

Responses to the CFSS-DS (Table 1) indicated that 53.7% and 55.4% of children were not afraid of dentists or doctors respectively, were only 24.8% and 19.0% slightly afraid of dentists or doctors. In contrast, 46.3% were very afraid of injections and 56.2% reported a high fear of choking while 55.4% of children were very afraid of being touched by a stranger. The results showed that 86.8% of children expressed no fear of people in white uniforms. The mean CFSS-DS total score was 36.7 ± 13.4, with 49.6% classified as anxious and 50.4% as non-anxious.

**Table 1:** Study participants’ responses to the Children’s Fear Survey Schedule – Dental Subscale

Are you afraid of	Not Afraid *N* (%)	Little Afraid *N* (%)	Fairly Afraid *N* (%)	Quite Afraid *N* (%)	Very Afraid *N* (%)
Dentists	65 (53.7)	30 (24.8)	15 (12.4)	1 (0.8)	10 (8.3)
Doctors	67 (55.4)	23 (19.0)	16 (13.2)	3 (2.5)	12 (9.9)
Injection (shots)	23 (19.0)	15 (12.4)	22 (18.2)	5 (4.1)	56 (46.3)
Having somebody examine your mouth	81 (66.9)	16 (13.2)	13 (10.7)	2 (1.7)	9 (7.4)
Having to open your mouth	71 (58.7)	14 (11.6)	18 (14.9)	3 (2.5)	15 (12.4)
Having a stranger touch you	19 (15.7)	16 (13.2)	13 (10.7)	6 (5.0)	67 (55.4)
Having somebody look at you	56 (46.3)	14 (11.6)	19 (15.7)	2 (1.7)	30 (24.8)
The dentist drilling	31 (25.6)	23 (19.0)	19 (15.7)	3 (2.5)	45 (37.2)
The sight of the dentist drilling	51 (42.1)	22 (18.2)	16 (13.2)	3 (2.5)	29 (24.0)
The noise of the dentist drilling	57 (47.1)	14 (11.6)	24 (19.8)	2 (1.7)	24 (19.8)
Having somebody put instrument in your mouth	37 (30.6)	26 (21.5)	15 (12.4)	5 (4.1)	38 (31.4)
Choking	16 (13.2)	18 (14.9)	14 (11.6)	5 (4.1)	68 (56.2)
Having to go to the hospital	72 (59.5)	13 (10.7)	19 (15.7)	5 (4.1)	12 (9.9)
People in white uniforms	105 (86.8)	2 (1.7)	7 (5.8)	1 (0.8)	6 (5.0)
Having the nurse clean your teeth	79 (65.3)	13 (10.7)	15 (12.4)	3 (2.5)	11 (9.1)

Entry-time analysis revealed that the participants generally fixated on instruments and operators more quickly in images without distractors than in images with distractors. Among anxious children, the mean entry time to instruments was significantly shorter in the no-distractor condition compared with the distractor condition (780.9 ± 700.1 ms vs. 2332.6 ± 1202.9 ms; *p* < 0.001). Similar patterns were observed for the handpiece and the operator’s eyes (both *p* < 0.05). In contrast, most of the other AOIs exhibited no significant differences between the anxious and non-anxious groups (Appendix B).

Dwell time analysis indicated longer fixations on clinical AOIs in non-distractor images than in those with distractors. Anxious participants devoted significantly more time fixating on instruments without distractors than with distractors (31.7% vs. 6.8%; *p* < 0.001). Comparable effects were observed for the dental chair, handpiece, and suction. Conversely, the distractors redirected their attention toward the screen (*p* < 0.001). Non-anxious participants had a significantly longer dwell time on instruments in image 4 than their anxious peers (*p* < 0.05) (Appendix C).

Sequence analysis revealed that AOIs were fixated on earlier and more consistently in the no-distractor images. With instruments as the AOI, 113 participants viewed the AOI second in the no-distractor condition compared with 51 in the distractor condition (*p* < 0.001). Similar differences in gaze order were found for the handpiece in all images (*p* < 0.05) (Appendix D).

Analysis of the number of AOI revisits further confirmed these trends. Around 115 participants revisited the instruments in the no-distractor condition and 52 in the distractor condition (*p* < 0.05). Suction AOI was also more frequent without distractors (53.7% vs. 39.8%; *p* < 0.05). Additionally, anxious children revisited the handpiece significantly fewer times than their non-anxious counterparts in the no-distractor condition (*p* < 0.05) (Appendix E).

**Fig. 6:**
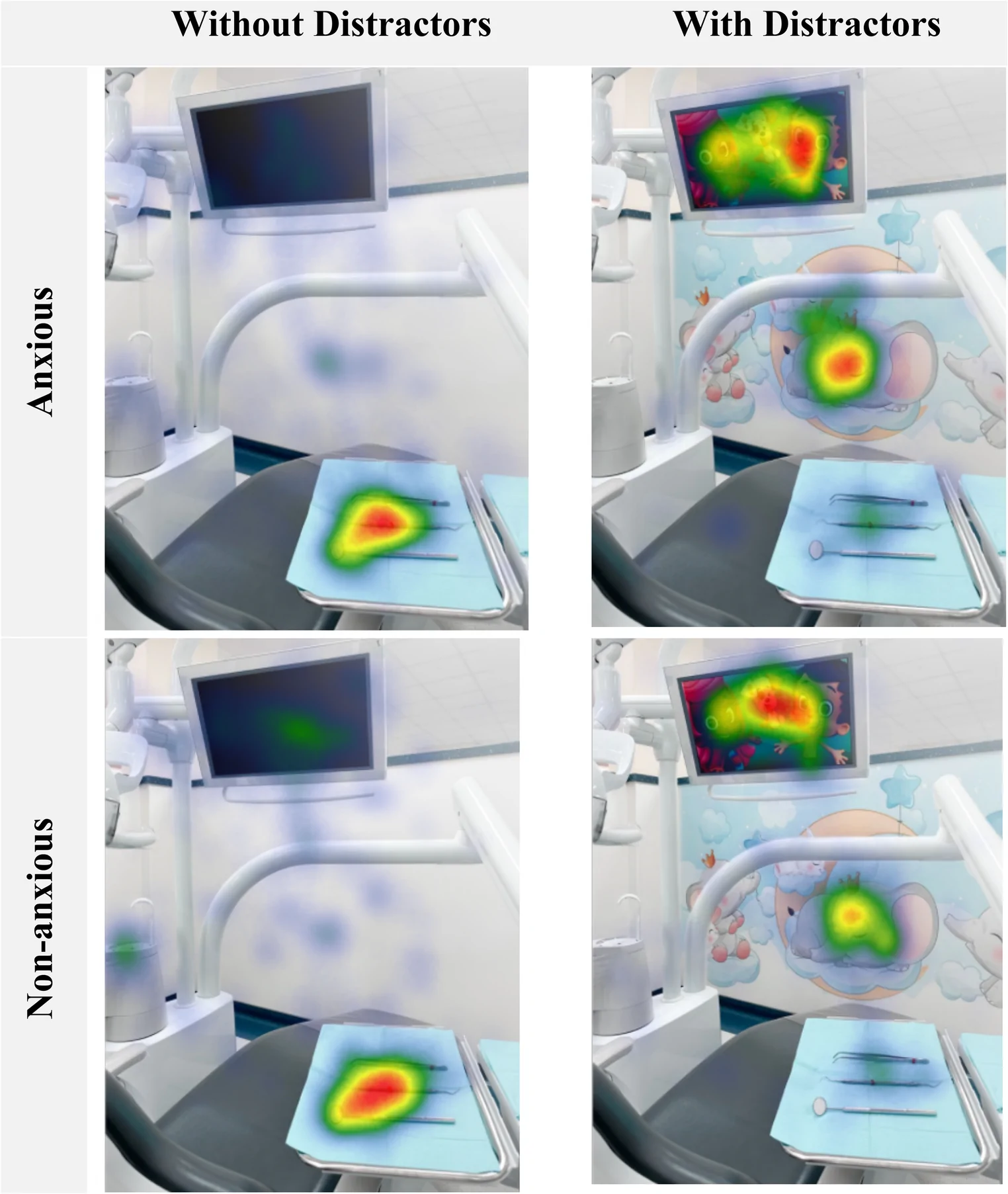
Dental Instruments’ view: focus of attention in anxious and non-anxious participants with and without distractors

In general, there was a high similarity between heat maps for anxious and non-anxious children. In the view displaying a part of the dental clinic and clearly showing the dental instruments (Fig. 6), both anxious and non-anxious children looked at instruments more frequently. Displaying the typical view image to the participants, but with added distractors (screen and stickers on the wall), demonstrated that the distractors succeeded in capturing participants’ attention from the instrument. However, anxious children looked at wall stickers and screens, whereas non-anxious children focused more on screens.

**Fig. 7:**
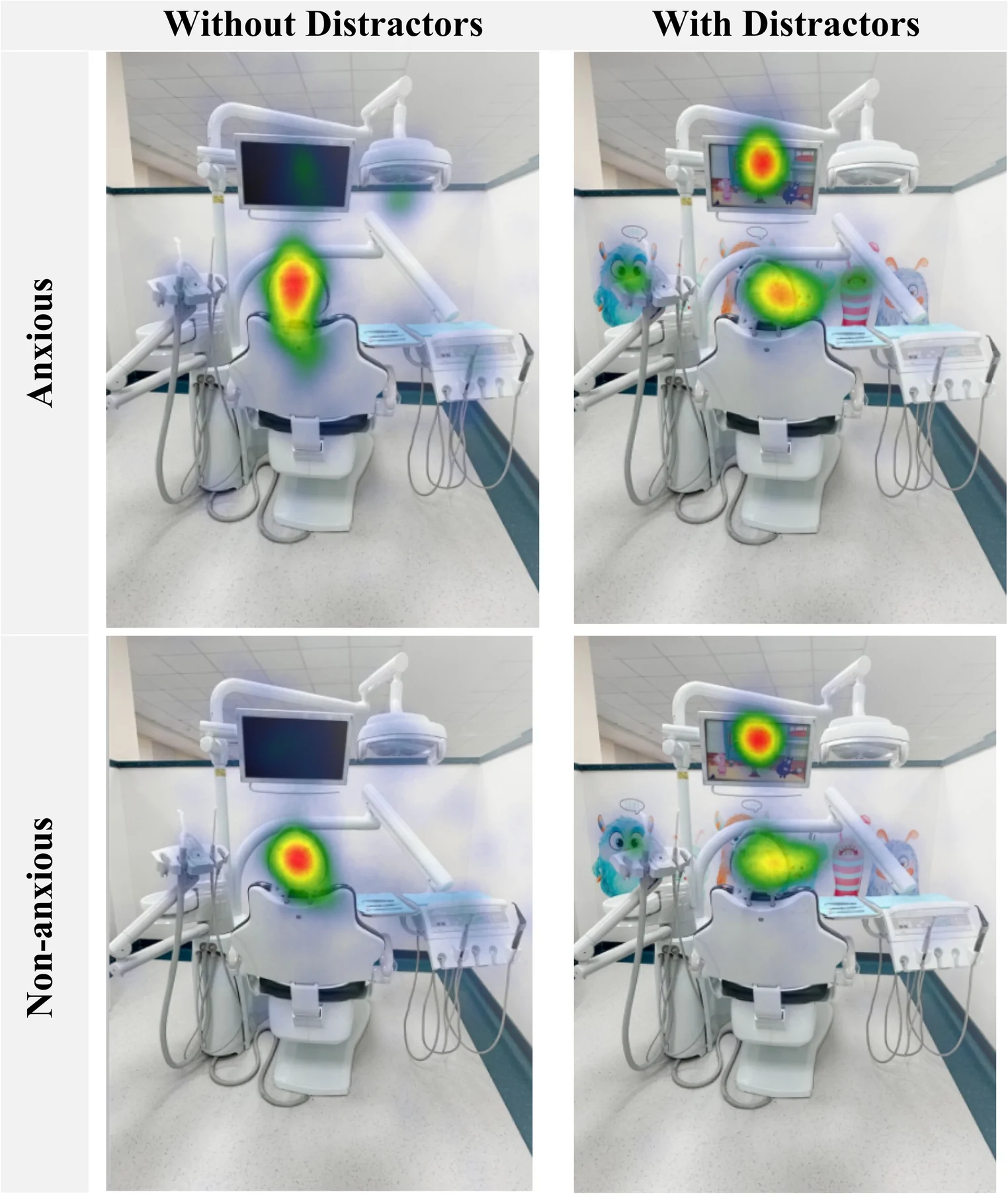
Dental clinic entrance’s view: focus of attention in anxious and non-anxious participants with and without distractors

In this view which resembles entering the clinic in a height of a child displaying the back of the dental chair (Fig. 7), in the pictures without distractors great similarities are shown between the heatmap of anxious and non-anxious children. Upon looking at the photos’ heatmap, participant’s focus was highly concentrated on the head of the dental chair and lightly on the rest of the dental unit. Displaying the other image with same view but with added distractors to the participants changed their way of looking at the image with a divided focus between the head of dental chair and the screen and slightly on the wall stickers.

**Fig. 8:**
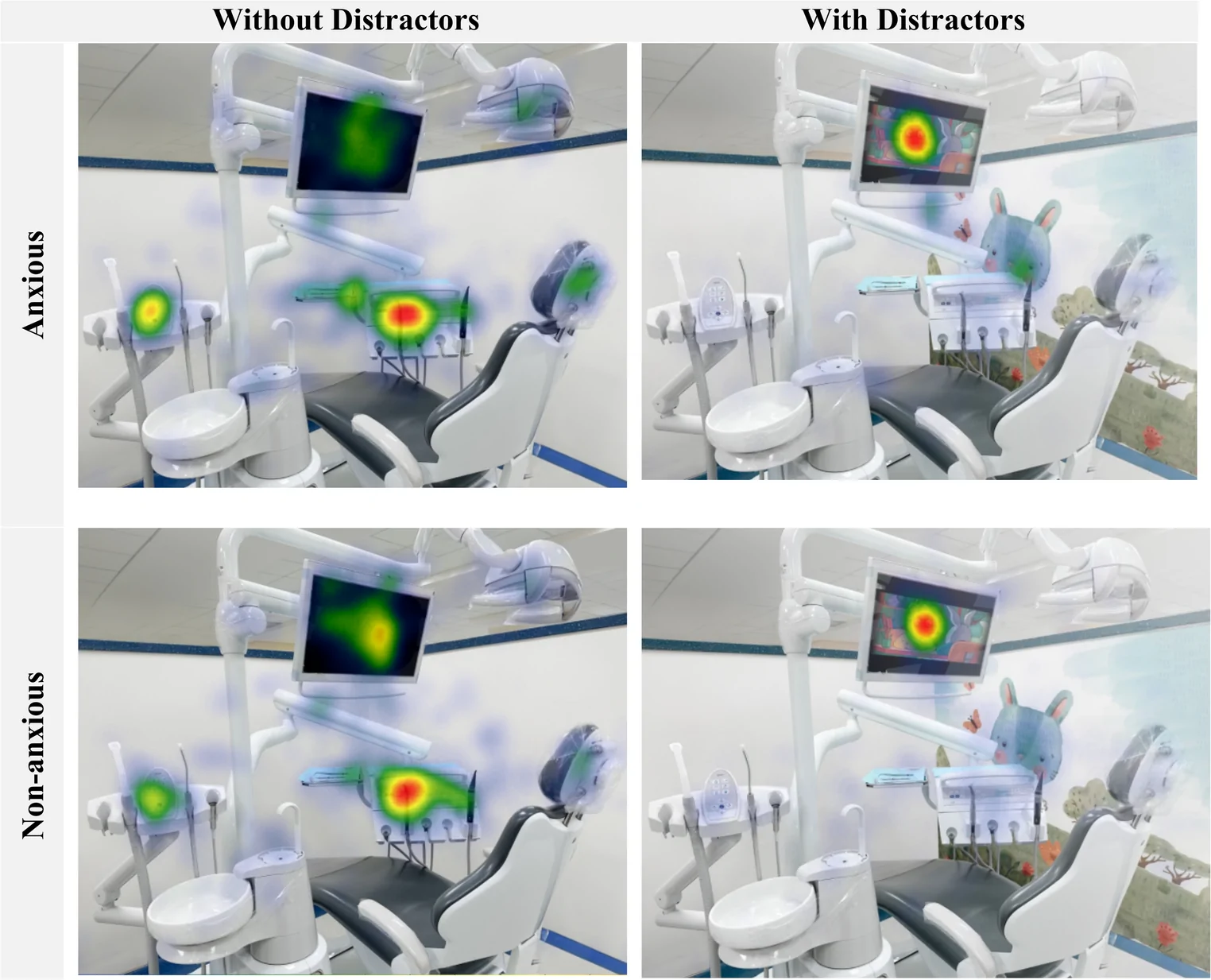
Dental unit side’s view: focus of attention in anxious and non-anxious participants with and without distractors

In a photograph where the dental unit is clearly displayed (Fig. 8), demonstrating a near view, attention of both groups in the images without distractors was greatly concentrated on the handpiece; however, it was followed by suction and then a black screen in the anxious group and, for the non-anxious group, followed by a black screen and, finally, suction, even though suction is not usually perceived as intimidating, it captured participants’ attention. Unexpectedly, both anxious and non-anxious groups partly concentrated on the screen, noting that it was turned off (black screen). However, when images with distractors were displayed to the children, their attention was mostly drawn to the screen displaying cartoons.

**Fig. 9:**
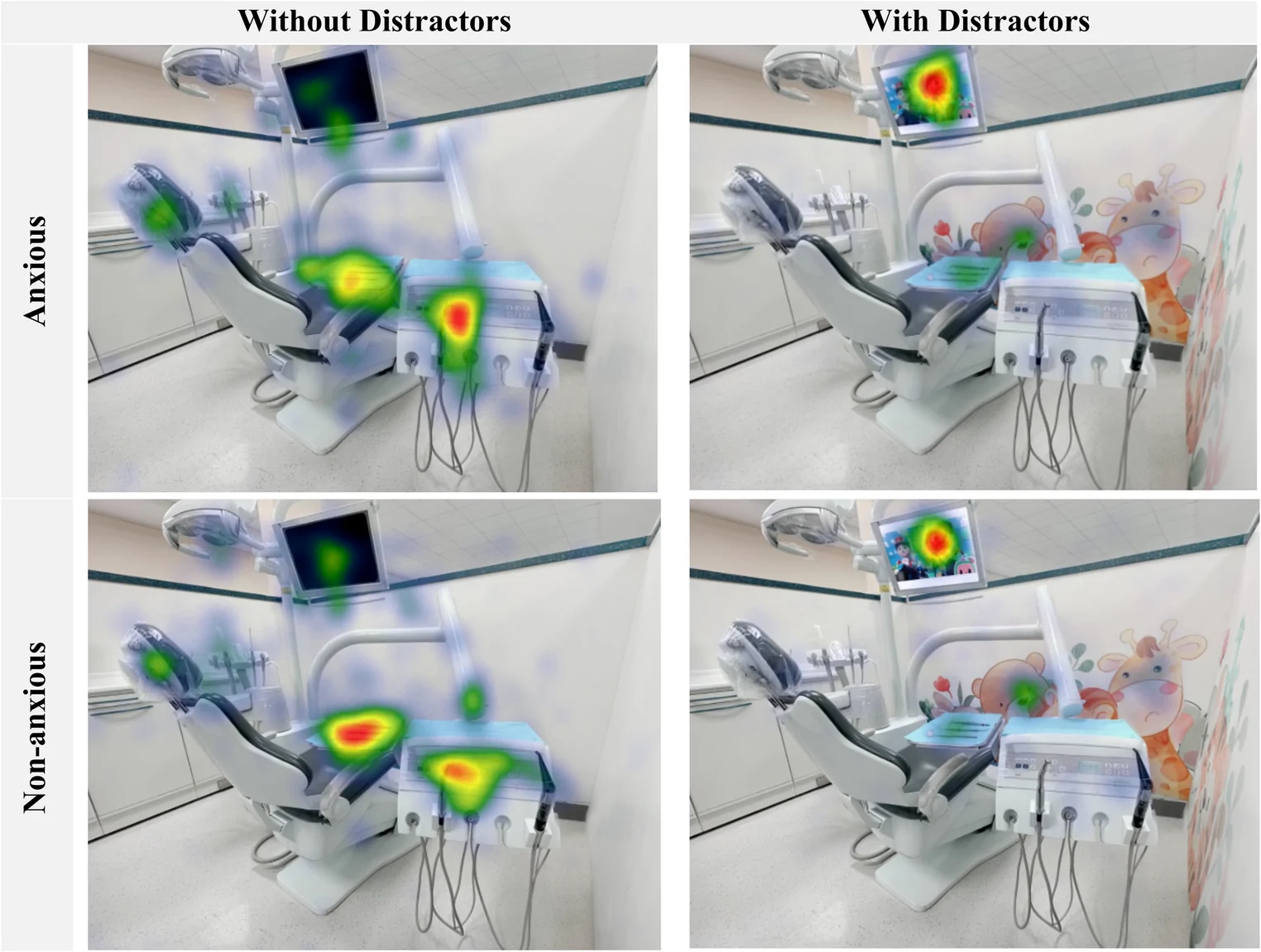
Dental unit side’s view with different angle: focus of attention in anxious and nonanxious participants with and without distractors

In the view demonstrating another side of the dental unit (Fig. 9) participant’s attention was mostly divided between handpiece and instruments and lightly on head of the dental chair, suction and black screen; however, when the same view with distractors was displayed to the participants their attention was almost completely drawn from the handpiece, instruments and head of dental chair and concentrated mostly on the screen displaying cartoons in both anxious and non-anxious groups.

**Fig. 10:**
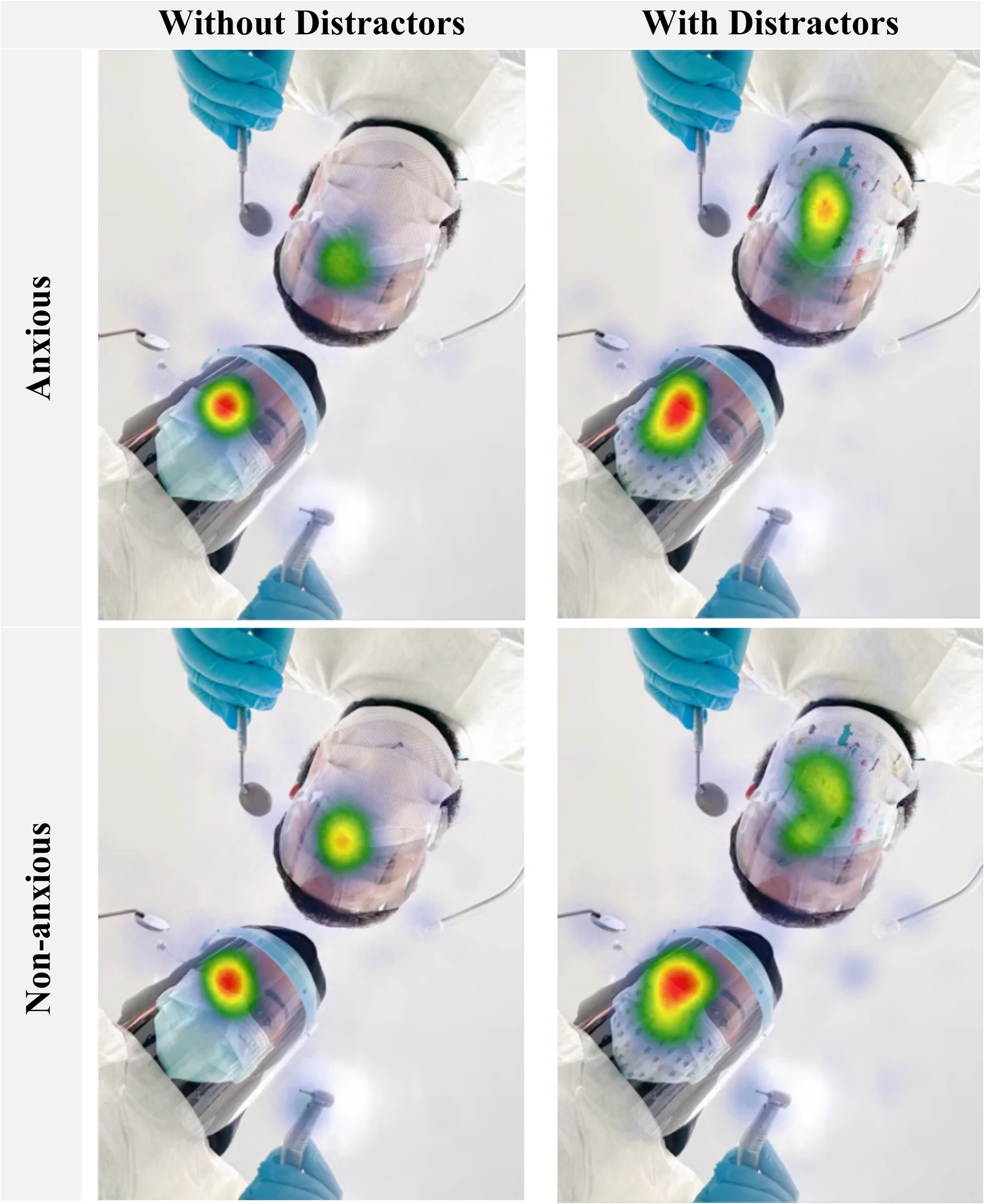
Patient’s view: attention focus of anxious and non-anxious participants with and without distractors on the dental team

In Fig. 10, which resembles the patient’s view when seated in the dental chair and is under examination or treatment by a dental team, the eyes of the operators grasped the participant’s attention in both anxious and non-anxious groups, while the instrument and the handpiece that might be intimidating did not capture the participant’s attention.

## Discussion

The present cross-sectional study evaluated the visual attention patterns of children 6–12 years of age in a dental operatory using eye-tracking technology by comparing anxious and non-anxious children, and assessed the effect of visual distractors, such as cartoons and wall stickers, on their gaze pattern(s). The results of this study provide a framework for understanding the influence of anxiety and environmental factors influence children during dental visits. This can help the dental team decide on the appropriate design of the pediatric dental operatory to provide a comfortable dental environment that will reduce anxiety among children and improve the quality of healthcare.

The age range of the participants (6–12 years) was appropriate for assessing DA because children at this stage can follow instructions while remaining susceptible to anxiety [8]. This range is consistent with earlier pediatric DA research that frequently targeted school-age children to capture both the behavioral and cognitive aspects of anxiety [9]. In the present study, participants were stratified as anxious or non-anxious using the validated CFSS-DS, a scale with established reliability and validity [3].

Nearly one-half of the participants scored above the CFSS-DS anxiety cut-off, despite most (90.9%) having previous dental visits. In the current study, injections and the sound/act of drilling were the most fear-provoking CFSS-DS items. Our findings are consistent with those of previous studies in which injections and dentist drilling [3], and pain- and noise-related items frequently ranked highest among children’s dental fears [20]. The mean score (36.7 ± 13.4) was consistent with findings from Kuwait and Saudi Arabia [21, 22], but higher than reports from Europe and North America [23]. Such variations underscore sociocultural influences on DA and the need for culturally tailored interventions. Although females were more often anxious, the difference was not significant, in contrast to studies reporting stronger sex effects in older cohorts [1, 24]. This suggests that sex differences may emerge more clearly after puberty, due to developmental or psychosocial factors.

In recent years, eye tracking technology has emerged as a powerful tool for analyzing visual attention and psychological responses in clinical, educational and experimental settings [15, 16, 25, 26]. In the present study, we used the SMI RED-m system, as in a study of gaze patterns among dental practitioners interpreting panoramic radiographs [27, 28]. Eye tracking provides novel insights into children’s attention to operatory stimuli. We analyzed gaze toward real operatory photographs by combining quantitative metrics (entry time, dwell time, sequence, and revisits) with qualitative heatmaps. To the best of our knowledge, this is the first application of the RED-m in pediatric dental anxiety research, stratifying patients into anxious and non-anxious groups.

Anxious children exhibited an “attention avoidance” pattern. In no-distractor conditions, they quickly oriented to threatening stimuli, such as instruments and the operator’s eyes; however, distractors significantly delayed entry time (e.g., instruments, 0.78 vs. 2.33 s; *p* < 0.001). This evidence supports that distraction can redirect attention away from clinical tools [12, 16] and may help reduce anxiety [11]. Similarly, dwell times for threatening AOIs decreased significantly when distractors were present, aligning with the avoidance responses described in childhood phobia research [29].

Sequence analysis further indicated that the distractors shifted initial fixations away from clinical instruments, interrupting the early processing of anxiety-provoking cues. This supports previous findings that visually engaging elements can alter attention flow in pediatric settings [16]. Additionally, revisit counts were lower in the distractor conditions, reflecting reduced sustained monitoring of threatening AOIs. This pattern parallels general anxiety research, in which revisits indicate ongoing threat evaluations [29]. Our findings suggest that distractors both capture initial attention and maintain engagement with non-threatening stimuli over time, which is consistent with evidence from distraction interventions in pediatric dentistry [16]. Anxious children’s shorter entry times and fewer revisits to the handpiece suggest initial vigilance, followed by avoidance of threatening stimuli. This aligns with pediatric phobia studies reporting rapid orientation and disengagement [29].

Two primary attention patterns were identified based on the results of this study. First, the avoidance pattern which was mainly observed in Anxious pediatric patients. Rapid orientation to threats (e.g., instruments) but low engagement/dwell time. Second, the Distraction Pattern which was observed in in both groups. When distractors were introduced, the attention was shifted, characterized by altered fixation sequences and redirected gaze density toward child-friendly elements (screens/stickers) and away from clinical tools.

It is important to note that 90.9% of our sample had previous dental visits. This high prevalence of past experience supports our interpretation of the “avoidance” pattern observed in anxious children. If the children had no prior multisensory experience, they might have explored the instruments with curiosity. Instead, the anxious group revisited the suction and handpieces significantly less frequently and spent less time looking at them than the non-anxious group. This specific avoidance behavior suggests they recognized the tools and associated them with negative past sensory experiences (e.g., noise or pain), consistent with our findings that “noise of the dentist drilling” and “injections” were high-ranking fear items on the CFSS-DS.

Heatmaps provide further evidence by illustrating the spatial concentrations of the gazes. Both anxious and non-anxious children focused heavily on clinical tools, such as handpieces, suction, and instrument tables in non-distractor conditions, which is consistent with previous findings [16]. These objects appeared to function as anchors of attention, underscoring their salience as anxiety triggers. When distractors were introduced, gaze density toward threatening AOIs was reduced, corroborating our quantitative results and confirming distraction effectiveness [12]. In simulated patient views, the operator’s eyes emerged as the primary focus for both groups, with anxious children devoting greater attention to decorated masks. This aligns with an earlier study demonstrating that children direct their gazes toward human faces in stressful contexts [25]. Facial cues remain powerful visual anchors during treatment, and attire or accessories can function as inadvertent distractors [25].

The present study had several strengths. Combining the validated CFSS-DS with high-resolution eye tracking enabled the simultaneous assessment of subjective fear levels and implicit attention behaviors. Within-picture manipulation reduced inter-individual variability and strengthened causal inferences regarding distractors. Using real clinical photographs enhanced ecological validity, with observed gaze shifts from instruments to cartoons or stickers, closely mirroring real-world settings. Eye-tracking minimizes self-report bias and provides quantifiable evidence of distraction effectiveness. Stratification according to anxiety status enabled a nuanced interpretation, demonstrating that anxious children distributed gazes across multiple distractors, whereas non-anxious children focused on a single salient element.

This study, however, also had some limitations that should be acknowledged. The single-center convenience sample restricts generalizability. In addition, anxiety expression and response to distractors could vary across cultures. Therefore, we recommend future studies to include participants across multiple clinical settings to enhance external validity of the results. The cross-sectional design prevented us from drawing conclusions regarding developmental progression. Future studies employing longitudinal designs are recommended to better explore developmental perspectives, changes in children’s responses over time, and changes in distracting effect using similar or different distractors. Priming effects may have occurred because of administering the CFSS-DS before eye tracking. Unmeasured confounders, such as parental anxiety, previous pain, and temperament, may have influenced the results. The restricted age range excluded adolescents, which limited developmental comparisons. Static images may not fully replicate real-time procedures, and gaze fixation reflects attention but not emotional engagement. Future studies incorporating physiological or behavioral measures or immersive virtual reality, could strengthen these interpretations. Finally, the use of 5-second image display time might be a limitation in this study. Although this duration was selected to allow the assessment of initial gaze orientation and visual attention patterns, yet it may not be sufficient for exploration of images containing multiple AOIs, particularly among children who may require additional processing time. Thus, this short display time may have favored early orientation responses and limited other metrics such as revisits. Additionally, longer display time may influence visual exploration patterns and consequently alter the observed avoidance pattern. Future studies should investigate the effect of varying image display time on pediatric gaze behavior.

Although static images provide a controlled and standardized method for assessing visual attention and gaze behavior, they may not fully replicate the complexity of real clinical dental environments, where children are exposed to dynamic interactions, sounds, movements, and emotional stimuli. Therefore, the generalizability of the present findings to actual clinical dental experiences should be interpreted with caution. Nevertheless, the visual attention patterns observed in this study may still have practical clinical implications, as incorporating child-friendly visual distractors within pediatric dental settings could help redirect children’s attention away from anxiety-provoking stimuli and potentially improve the overall dental experience. Additionally, the inclusion of a broad developmental age range (6–12 years) without age-stratified analysis may have obscured potential age-related differences in gaze behavior and response to distractors, given the variability in cognitive and attentional abilities across these ages. Future research should investigate visual attention behavior using more dynamic and immersive methodologies, such as video-based stimuli, virtual reality environments, or real-time clinical eye-tracking assessments, and should incorporate age-stratified analyses to better understand developmental differences in pediatric gaze behavior and anxiety responses in dental settings.

Overall, the findings indicated that visual distractors significantly influenced gaze behavior in both anxious and non-anxious children. Without distractors, anxious participants exhibited shorter entry times to the handpiece, shorter dwell times on instruments, and fewer revisits to the handpiece than their non-anxious peers. When distractors were introduced, between-group differences diminished but gaze patterns consistently shifted away from threatening instruments toward more child-friendly elements. These results highlight the effectiveness of distraction in modifying visual attention and suggest that anxiety-related differences may be more pronounced in the absence of such interventions.

## Conclusion

This study contributes to the design of anxiety-reducing pediatric dental environments by identifying elements that attract the visual attention of children. The findings of this study revealed that anxious children exhibited faster orientation but shorter engagement with dental instruments, particularly in the absence of distractors. Thus, the presence of appropriate distractors within the pediatric dental clinic may help redirect children’s gaze away from clinical tools and instruments. Introducing cartoon-based screens and wall stickers effectively redirected attention away from threatening stimuli toward more child-friendly elements, underscoring the role of the design and layout of pediatric dental clinics in improving the child’s comfort and cooperation. Consistent visual focus on clinicians’ faces, especially the eyes, also emphasizes the importance of provider demeanor. These findings highlight the potential of targeted environmental modifications combined with attentive provider behavior to reduce anxiety and improve the overall child’s experience in the pediatric dental clinics.

## Data Availability

The datasets of this study are available from the corresponding author on reasonable request.

## Appendix B

**Table:** Participants’ Entry Time to the areas of interests [ms], *N* = 121

Picture	AOI	Distractors	Anxious *n* = 60	Non-anxious *n* = 61	*p*-value §	All subjects *n* = 121
	n (%) Mean ± SD Median (Interquartile Range)					n (%) Mean ± SD Median (Interquartile Range)
One	**Instruments**	**No distractor**	57 (95.0) 780.9 ± 700.1 585 (438–804)	58 (95.1) 808.3 ± 811.5 561 (406–803)	0.810	115 (95.0) 794.7 ± 755.1 574 (413–786)
		**With distractor**	28 (46.7) 2332.6 ± 1202.9 585 (442–786)	24 (39.3) 1918.4 ± 1037.7 561 (407–774)	0.279	52 (43.0) 2141.5 ± 1138.0 1806 (1260–2996)
		***p*** **-value §**				
			**< 0.001***	**< 0.001***		**< 0.001***
Two	**Dental chair (Back)**	**No distractor**	60 (100) 416.8 ± 507.3 276 (198–449)	58 (95.1) 690.2 ± 985.3 341 (231–530)	0.167	118 (97.5) 551.2 ± 788.4 300 (222–502)
		**With distractor**	58 (96.7) 360.8 ± 367.5 266 (206–397)	57 (93.4) 426.8 ± 613.9 274 (177–430)	0.929	115 (95.0) 393.5 ± 503.8 268 (199–413)
		***p*** **-value §**	0.577	0.100		0.129
Three	**Handpiece**	**No distractor**	21 (35.0) 1801.4 ± 1371.1 1601 (570–2900)	22 (36.1) 1759.7 ± 1376.6 1401 (383–2842)	1.00	43 (35.5) 1780.1 ± 1357.6 1601 (522–2873)
		**With distractor**	3 (5.0) 4068.4 ± 247.9 4044 (2833–4185)	9 (14.8) 2159.1 ± 1555.9 1372 (892–3506)	0.064	12 (9.9) 2636.4 ± 1586.7 3094 (1119–3991)
		***p*** **-value §**	**0.031***	0.480		0.091
	**Suction**	**No distractor**	33 (55.0) 2172.8 ± 1093.3 1956 (1401–2800)	32 (52.5) 2134.8 ± 1143.6 1864 (1375–3050)	0.990	65 (53.7) 2154.1 ± 1109.7 1956 (1401–3013)
		**With distractor**	16 (26.7) 3013.1 ± 1255.6 3276 (1940–4121)	20 (32.8) 3032.2 ± 994.6 3164 (2081–3741)	0.912	36 (39.8) 3023.7 ± 1101.3 3196 (2035–3894)
		***p*** **-value §**	**0.028***	**0.006***		**< 0.001***
	**Screen**	**No distractor**	57 (95.0) 1022.4 ± 1049.3 607 (281–1465)	53 (86.9) 841.9 ± 1125.2 441 (14–1259)	0.179	110 (90.9) 935.5 ± 1085.3 508 (15–1299)
		**With distractor**	58 (96.7) 622.2 ± 525.7 495 (328–715)	60 (98.4) 565.7 ± 622.5 457 (322–629)	0.398	118 (97.5) 593.5 ± 575.2 470 (326–653)
		***p*** **-value §**	0.181	0.904		0.329
Four	**Instruments**	**No distractor**	22 (36.7) 1805.2 ± 1684.9 1305 (296–3153)	35 (57.4) 1429.9 ± 1278.8 847 (328–2700)	0.870	57 (47.1) 1574.7 ± 1446.1 908 (315–2825)
		**With distractor**	26 (43.3) 2189.3 ± 1297.9 2249 (1502–2935)	22 (36.1) 1979.8 ± 1283.2 2084 (988–2934)	0.591	48 (39.7) 2093.3 ± 1281.8 2105 (1251–2910)
		***p*** **-value §**	0.444	0.140		0.079
	**Handpiece**	**No distractor**	9 (15.0) 1812.1 ± 1082.8 1729 (736–2815)	11 (18.0) 2577.7 ± 1572.7 2820 (1148–4030)	0.370	20 (16.5) 2233.2 ± 1395.8 1799 (1106–3368)
		**With distractor**	2 (3.3) 2545.3 ± 1771.7 2545 (1292–3798)	2 (3.3) 4118.5 ± 73.5 4118 (4066–4170)	0.333	4 (3.3) 3331.9 ± 1368.6 3932 (1918–4144)
		***p*** **-value §**	0.582	0.231		0.157
Five	**Mirror**	**No distractor**	14 (23.3) 2564.3 ± 1403.4 2401 (1446–3463)	11 (18.0) 3350.6 ± 1367.7 3269 (2949–4410)	0.134	25 (20.7) 2910.3 ± 1415.9 2972 (1926–4343)
		**With distractor**	11 (18.0) 2509.9 ± 1720.4 2886 (893–4192)	13 (21.3) 2848.9 ± 1429.8 2606 (2015–4250)	0.776	24 (19.8) 2693.6 ± 1543.7 2725 (1600–4120)
		***p*** **-value §**	0.893	0.228		0.603
	**Handpiece**	**No distractor**	13 (21.7) 3295.1 ± 1068.1 2549 (1514–3322)	18 (29.5) 3378.2 ± 1097.8 3654 (2825–4170)	**0.012***	31 (25.6) 2965.9 ± 1175.8 3245 (2241–3954)
		**With distractor**	9 (15.0) 3064.7 ± 1103.3 3220 (2144–3943)	8 (13.1) 2334.3 ± 957.4 2696 (1275–3182)	0.139	17 (14.0) 2720.9 ± 1072.8 3004 (1351–3565)
		***p*** **-value §**	0.164	**0.022***		0.337
	**Suction**	**No distractor**	2 (3.3) 4289.1 ± 792.0 4289 (3729–4849)	5 (8.2) 3732.0 ± 1119.3 4094 (2959–4658)	0.571	7 (5.8) 3891.2 ± 1006.8 4094 (2959–4756)
		**With distractor**	2 (3.3) 3634.6 ± 1607.8 3634 (2497–4771)	4 (6.6) 3814.8 ± 796.2 4038 (3343–4286)	1.00	6 (5.0) 3754 ± 951.9 4038 (2626–4578)
		***p*** **-value §**	0.667	0.730		0.731
	**Eyes**	**No distractor**	47 (78.3) 1677.2 ± 753.0 1505 (1152–1871)	54 (88.5) 1919.2 ± 1169.3 1606 (1046–2475)	0.653	101 (83.5) 1806.6 ± 1000.1 1523 (1126–2320)
		**With distractor**	28 (46.7) 3302.3 ± 1891.5 3277 (1502–4702)	30 (49.2) 2548.9 ± 1684.3 2044 (1182–3781)	0.102	58 (47.9) 2912.7 ± 1811.7 2562 (1374–4211)
		***p*** **-value §**	**< 0.001***	0.173		**< 0.001***

§ Mann Whitney U test

*n* number of participants

*Statistically significant (*p* < 0.05)

## Appendix C

**Table:** Participants’ Dwell Time at the areas of interests [%], *N* = 121

Picture	AOI	Distractors	Anxious *n* = 60	Non-anxious *n* = 61	*p*-value §	All subjects *n* = 121
	Mean ± SD Median (Interquartile Range)					Mean ± SD Median (Interquartile Range)
One	**Instruments**	**No distractor**	31.7 ± 18.5 30 (17–43)	32.0 ± 20.3 30 (15–46)	0.928	31.8 ± 19.4 30 (16–44)
		**With distractor**	6.8 ± 11.2 0 (0–8)	7.3 ± 15.7 0 (0–8)	0.585	7.1 ± 13.6 0 (0–8)
		***p*** **-value ¥**	**< 0.001***	**< 0.001***		**< 0.001***
Two	**Dental chair (Back)**	**No distractor**	36.2 ± 19.8 33 (20–50)	33.2 ± 21.8 29 (20–41)	0.320	34.6 ± 20.8 29 (20–41)
		**With distractor**	26.3 ± 15.4 24 (15–33)	26.1 ± 13.9 26 (18–34)	0.611	26.2 ± 14.6 25 (17–34)
		***p*** **-value ¥**	**0.002***	0.101		**< 0.001***
Three	**Handpiece**	**No distractor**	3.5 ± 6.5 0 (0–6.7)	3.4 ± 6.3 0 (0–5.7)	0.937	3.4 ± 6.4 0 (0–6)
		**With distractor**	0.4 ± 1.9 0 (0–0)	1.2 ± 4.1 0 (0–0)	0.079	0.8 ± 3.3 0 (0–0)
		***p*** **-value ¥**	**< 0.001***	**0.007***		**< 0.001***
	**Suction**	**No distractor**	6.8 ± 8.0 5.7 (0–11)	7.2 ± 9.2 3.3 (0–11)	0.952	7.0 ± 8.6 4 (0–11)
		**With distractor**	2.3 ± 4.5 0 (0–1)	3.2 ± 6.4 0 (0–3)	0.465	2.8 ± 5.6 0 (0–3)
		***p*** **-value ¥**	**< 0.001***	**0.001***		**< 0.001***
	**Screen**	**No distractor**	16.3 ± 11.1 14 (8–23)	18.7 ± 19.9 15 (5–23)	0.620	17.5 ± 16.1 14 (7–23)
		**With distractor**	34.5 ± 20.7 32 (19–44)	37.9 ± 20.3 36 (22–48)	0.305	36.2 ± 20.5 34 (20–47)
		***p*** **-value ¥**	**< 0.001***	**< 0.001***		**< 0.001***
Four	**Instruments**	**No distractor**	3.6 ± 5.9 0 (0–6)	7.9 ± 10.6 5 (0–12)	**0.011***	5.8 ± 8.8 0 (0–9)
		**With distractor**	5.9 ± 10.5 0 (0–8)	5.7 ± 11.2 0 (0–9)	0.542	5.8 ± 10.8 0 (0–8)
		***p*** **-value ¥**	0.334	0.104		0.546
	**Handpiece**	**No distractor**	2.1 ± 5.9 0 (0–0)	2.4 ± 6.9 0 (0–0)	0.698	2.3 ± 6.4 0 (0–0)
		**With distractor**	0.6 ± 3.6 0 (0–0)	0.2 ± 1.3 0 (0–0)	0.973	0.4 ± 2.7 0 (0–0)
		***p*** **-value ¥**	0.062	**0.017***		**0.002***
Five	**Mirror**	**No distractor**	2.0 ± 4.7 0 (0–0)	1.3 ± 3.2 0 (0–0)	0.498	1.7 ± 4.0 0 (0–0)
		**With distractor**	1.9 ± 5.1 0 (0–0)	1.8 ± 4.7 0 (0–0)	0.752	1.9 ± 4.9 0 (0–0)
		***p*** **-value ¥**	0.770	0.702		0.952
	**Handpiece**	**No distractor**	1.6 ± 3.5 0 (0–0)	3.3 ± 6.7 0 (0–4)	0.224	2.5 ± 5.4 0 (0–2)
		**With distractor**	1.3 ± 3.4 0 (0–0)	1.4 ± 3.9 0 (0–0)	0.830	1.3 ± 3.6 0 (0–0)
		***p*** **-value ¥**	0.601	0.061		0.071
	**Suction**	**No distractor**	0.2 ± 1.4 0 (0–0)	0.4 ± 1.4 0 (0–0)	0.256	0.3 ± 1.4 0 (0–0)
		**With distractor**	0.2 ± 1.2 0 (0–0)	0.6 ± 2.5 0 (0–0)	0.393	0.4 ± 1.9 0 (0–0)
		***p*** **-value ¥**	1.00	0.441		0.504
	**Eyes**	**No distractor**	44.1 ± 23.1 45 (22–61)	46.9 ± 21.4 48 (32–62)	0.561	45.6 ± 22.2 48 (28–61)
		**With distractor**	19.8 ± 16.0 17 (9–25)	23.5 ± 15.5 21 (10–36)	0.108	21.7 ± 15.8 19 (9–31)
		***p*** **-value ¥**	**< 0.001***	**< 0.001***		**< 0.001***

§ Mann Whitney U test

*n* number of participants

*Statistically significant (*p* < 0.05)

## Appendix D

**Table:** Participants’ Sequence Table, *N* = 121

Picture	AOI	Distractors		Anxious *n* = 60	Non-anxious *n* = 61	*p*-value €	All subjects *n* = 121
	n (%)						n (%)
1	**Instruments**	**No distractor**	**1**	2 (3.3)	0	0.355	2 (1.7)
			**2**	55 (91.7)	58 (95.1)		113 (93.4)
			**OL**	3 (5.0)	3 (4.9)		6 (5.0)
		**With distractor**	**1**	0	1 (1.6)	0.398	1 (0.8)
			**2**	28 (46.7)	23 (37.7)		51 (42.1)
			**OL**	32 (53.3)	37 (60.7)		69 (57.0)
		***p*** **-value ₣**		**< 0.001***	**< 0.001***		**< 0.001***
2	**Dental chair (Back)**	**No distractor**	**1**	15 (25.0)	10 (16.4)	0.129	25 (20.7)
			**2**	45 (75.0)	48 (78.7)		93 (76.9)
			**OL**	0	3 (4.9)		3 (2.5)
		**With distractor**	**1**	24 (40.0)	23 (37.7)	0.712	47 (38.8)
			**2**	34 (56.7)	34 (55.7)		68 (56.2)
			**OL**	2 (3.3)	4 (6.6)		6 (5.0)
		***p*** **-value ₣**		**0.014***	**0.012***		**< 0.001***
3	**Handpiece**	**No distractor**	**1**	1 (1.7)	0	0.882	1 (0.8)
			**2**	8 (13.3)	8 (13.1)		16 (13.2)
			**3**	10 (16.7)	12 (19.7)		22 (18.2)
			**4**	2 (3.3)	2 (3.3)		4 (3.3)
			**OL**	39 (65.0)	39 (63.9)		78 (64.5)
		**With distractor**	**1**	0	0	0.106	0
			**2**	0	2 (3.3)		2 (1.7)
			**3**	1 (1.7)	6 (9.8)		7 (5.8)
			**4**	2 (3.3)	1 (1.6)		3 (2.5)
			**OL**	57 (95.0)	52 (85.2)		109 (90.1)
		***p*** **-value ₣**		**< 0.001***	**0.019***		**< 0.001***
	**Suction**	**No distractor**	**1**	0	0	0.315	0
			**2**	6 (10.0)	12 (19.7)		18 (14.9)
			**3**	20 33.3()	13 (21.3)		33 (27.3)
			**4**	7 (11.7)	7 (11.5)		14 (11.6)
			**OL**	27 (45.0)	29 (47.5)		56 (46.3)
		**With distractor**	**1**	0	0	0.238	0
			**2**	1 (1.7)	0		1 (0.8)
			**3**	15 (25.0)	17 (27.9)		32 (26.4)
			**4**	0	3 (4.9)		3 (2.5)
			**OL**	44 (73.3)	41 (67.2)		85 (70.2)
		***p*** **-value ₣**		**0.009***	0.303		**0.010***
	**Screen**	**No distractor**	**1**	13 (21.7)	19 (31.1)	0.270	32 (26.4)
			**2**	33 (55.0)	23 (37.7)		56 (46.3)
			**3**	10 (16.7)	10 (16.4)		20 (16.5)
			**4**	1 (1.7)	1 (1.6)		2 (1.7)
			**OL**	3 (5.0)	8 (13.1)		11 (9.1)
		**With distractor**	**1**	5 (8.3)	8 (13.1)	0.708	13 (10.7)
			**2**	52 (86.7)	50 (82.0)		102 (84.3)
			**3**	1 (1.7)	2 (3.3)		3 (2.5)
			**OL**	2 (3.3)	1 (1.6)		3 (2.5)
		***p*** **-value ₣**		1.00	0.160		0.292
4	**Instruments**	**No distractor**	**1**	2 (3.3)	1 (1.6)	0.100	3 (2.5)
			**2**	18 (30.0)	30 (49.2)		48 (39.7)
			**3**	2 (3.3)	4 (6.6)		6 (5.0)
			**OL**	38 (63.3)	26 (42.6)		64 (52.9)
		**With distractor**	**1**	1 (1.7)	4 (6.6)	0.262	5 (4.1)
			**2**	24 (40.0)	18 (29.5)		42 (34.7)
			**3**	1 (1.7)	0		1 (0.8)
			**OL**	34 (56.7)	39 (63.9)		73 (60.3)
		***p*** **-value ₣**		0.622	**< 0.001***		0.056
	**Handpiece**	**No distractor**	**1**	1 (1.7)	0	0.423	1 (0.8)
			**2**	7 (11.7)	7 (11.5)		14 (11.6)
			**3**	1 (1.7)	4 (6.6)		5 (4.1)
			**OL**	51 (85.0)	50 (82.0)		101 (83.5)
		**With distractor**	**1**	0	0	0.513	0
			**2**	1 (1.7)	2 (3.3)		3 (2.5)
			**3**	1 (1.7)	0		1 (0.8)
			**OL**	58 (96.7)	59 (96.7)		117 (96.7)
		***p*** **-value ₣**		**0.035***	**0.013***		**0.001***
5	**Mirror**	**No distractor**	**1**	1 (1.7)	1 (1.6)	0.292	2 (1.7)
			**2**	0	0		0
			**3**	5 (8.3)	1 (1.6)		6 (5.0)
			**4**	7 (11.7)	5 (8.2)		12 (9.9)
			**5**	1 (1.7)	4 (6.6)		5 (4.1)
			**OL**	46 (76.7)	50 (82.0)		96 (79.3)
		**With distractor**	**1**	2 (3.3)	1 (1.6)	0.830	3 (2.5)
			**2**	5 (8.3)	5 (8.2)		10 (8.3)
			**3**	3 (5.0)	4 (6.6)		7 (5.8)
			**4**	1 (1.7)	3 (4.9)		4 (3.3)
			**OL**	49 (81.7)	48 (78.7)		97 (80.2)
		***p*** **-value ₣**		0.275	0.827		0.355
	**Handpiece**	**No distractor**	**1**	1 (1.7)	0	0.461	1 (0.8)
			**2**	0	0		0
			**3**	5 (8.3)	4 (6.6)		9 (7.4)
			**4**	6 (10.0)	12 (19.7)		18 (14.9)
			**5**	1 (1.7)	2 (3.3)		3 (2.5)
			**OL**	47 (78.3)	43 (70.5)		90 (74.4)
		**With distractor**	**1**	0	0	0.794	0
			**2**	3 (5.0)	3 (4.9)		6 (5.0)
			**3**	5 (8.3)	5 (8.2)		10 (8.3)
			**4**	0	0		0
			**5**	1 (1.7)	0		1 (0.8)
			**OL**	51 (85.0)	53 (86.9)		104 (86.0)
		***p*** **-value ₣**		0.346	**0.007***		**0.008***
	**Suction**	**No distractor**	**1**	0	0	0.359	0
			**2**	0	0		0
			**3**	0	1 (1.6)		1 (0.8)
			**4**	0	2 (3.3)		2 (1.7)
			**5**	2 (3.3)	1 (1.6)		3 (2.5)
			**6**	0	1 (1.6)		1 (0.8)
			**OL**	58 (96.7)	56 (91.8)		114 (94.2)
		**With distractor**	**1**	0	0	0.721	0
			**2**	0	1 (1.6)		1 (0.8)
			**3**	0	0		0
			**4**	1 (1.7)	1 (1.6)		2 (1.7)
			**5**	1 (1.7)	2 (3.3)		3 (2.5)
			**OL**	58 (96.7)	57 (93.4)		115 (95.0)
		***p*** **-value ₣**		0.546	1.00		0.763

*OL* Overlooked

*n* number of participants

₣ Related Sample Friedman test

€ chi-square test

*Statistically significant (*p* < 0.05)

## Appendix E

**Table:** Participants’ Revisits to the areas of interests, *N* = 121

Picture	AOI	Distractors	Anxious *n* = 60	Non-anxious *n* = 61	*p*-value §	All subjects *n* = 121
	n (%) Median (Minimum - Maximum)					n (%) Median (Minimum - Maximum)
One	**Instruments**	**No distractor**	57 (95.0) 1 (0–3)	58 (95.1) 0 (0–4)	0.173	115 (95.0) 1 (0–4)
		**With distractor**	28 (46.7) 0 (0–2)	24 (39.3) 0 (0–2)	0.767	52 (43.0) 0 (0–2)
		***p*** **-value §**	**0.015***	0.389		**0.018***
Two	**Dental chair (Back)**	**No distractor**	60 (100) 1 (0–5)	58 (95.1) 1 (0–4)	0.742	118 (97.5) 1 (0–5)
		**With distractor**	58 (96.7) 2 (0–4)	57 (93.4) 2 (0–4)	0.503	115 (95.0) 2 (0–4)
		***p*** **-value §**	0.187	0.121		**0.041***
Three	**Handpiece**	**No distractor**	21 (35.0) 0 (0–1)	22 (36.1) 0 (0–1)	**0.035***	43 (35.5) (0–1)
		**With distractor**	3 (5.0) 0 (0–0)	9 (14.8) 0 (0–1)	0.600	12 (9.9) 0 (0–1)
		***p*** **-value §**	0.452	0.453		0.975
	**Suction**	**No distractor**	33 (55.0) 0 (0–1)	32 (52.5) 0 (0–2)	0.672	65 (53.7) 0 (0–2)
		**With distractor**	16 (26.7) 0 (0–1)	20 (32.8) 0 (0–1)	0.718	36 (39.8) 0 (0–1)
		***p*** **-value §**	0.250	**0.025***		**0.015***
	**Screen**	**No distractor**	57 (95.0) 1 (0–4)	53 (86.9) 1 (0–5)	0.861	110 (90.9) 1 (0–5)
		**With distractor**	58 (96.7) 1 (0–3)	60 (98.4) 1 (0–4)	0.446	118 (97.5) 1 (0–4)
		***p*** **-value §**	0.944	0.388		0.517
Four	**Instruments**	**No distractor**	22 (36.7) 0 (0–2)	35 (57.4) 0 (0–2)	0.523	57 (47.1) 0 (0–2)
		**With distractor**	26 (43.3) 0 (0–2)	22 (36.1) 0 (0–3)	0.651	48 (39.7) 0 (0–3)
		***p*** **-value §**	0.770	0.692		0.516
	**Handpiece**	**No distractor**	9 (15.0) 0 (0–1)	11 (18.0) 0 (0–1)	0.824	20 (16.5) 0 (0–1)
		**With distractor**	2 (3.3) 0 (0–0)	2 (3.3) 0 (0–0)	1.00	4 (3.3) 0 (0–0)
		***p*** **-value §**	0.909	0.769		0.682
Five	**Mirror**	**No distractor**	14 (23.3) 0 (0–1)	11 (18.0) 0 (0–1)	0.809	25 (20.7) 0 (0–1)
		**With distractor**	11 (18.0) 0 (0–1)	13 (21.3) 0 (0–2)	0.955	24 (19.8) 0 (0–2)
		***p*** **-value §**	0.893	0.691		0.587
	**Handpiece**	**No distractor**	13 (21.7) 0 (0–1)	18 (29.5) 0 (0–1)	0.594	31 (25.6) 0 (0–1)
		**With distractor**	9 (15.0) 0 (0–0)	8 (13.1) 0 (0–1)	0.423	17 (14.0) 0 (0–1)
		***p*** **-value §**	0.393	0.605		0.685
	**Suction**	**No distractor**	2 (3.3) 0 (0–0)	5 (8.2) 0 (0–0)	1.00	7 (5.8) 0 (0–0)
		**With distractor**	2 (3.3) 0 (0–0)	4 (6.6) 0 (0–0)	1.00	6 (5.0) 0 (0–0)
		***p*** **-value §**	1.00	1.00		1.00

§ Mann Whitney U test

*n* number of participants

*Statistically significant (*p* < 0.05)

## References

[1] Seligman LD, Hovey JD, Chacon K, Ollendick TH (2017) Dental anxiety: An understudied problem in youth. Clin Psychol Rev 55:25–40 28478271 10.1016/j.cpr.2017.04.004

[2] Aartman IH, van Everdingen T, Hoogstraten J, Schuurs AH (1998) Self-report measurements of dental anxiety and fear in children: a critical assessment. ASDC J Dent Child 65(4):252–258 9740944

[3] El-Housseiny AA, Alsadat FA, Alamoudi NM, El Derwi DA, Farsi NM, Attar MH et al (2016) Reliability and validity of the children’s fear survey schedule-dental subscale for Arabic-speaking children: a cross-sectional study. BMC Oral Health 16:49 27079656 10.1186/s12903-016-0205-0 PMC4832529

[4] Kvesić AJ, Hrelja M, Lovrić Ž, Šimunović L, Špiljak B, Supina N et al (2023) Possible risk factors for dental fear and anxiety in children who suffered traumatic dental injury. Dent J (Basel) 11(8):190 37623286 10.3390/dj11080190 PMC10453853

[5] Paiva ACF, Rabelo-Costa D, Fernandes IB, Magno MB, Maia LC, Paiva SM et al (2023) The relationship between temperament and dental fear and anxiety: a systematic review. Evid Based Dent 24(1):42 36869119 10.1038/s41432-023-00852-0

[6] Shindova MP, Belcheva AB (2021) Dental fear and anxiety in children: a review of the environmental factors. Folia Med (Plovdiv) 63(2):177–182 33932006 10.3897/folmed.63.e54763

[7] Uzel İ, Aydınel B, Ak AT (2022) Evaluation of the risk factors of dental anxiety in children. J Pediatr Res 9:99–104

[8] Kakkar M, Wahi A, Thakkar R, Vohra I, Shukla AK (2016) Prevalence of dental anxiety in 10–14 years old children and its implications. J Dent Anesth Pain Med 16(3):199–202 28884153 10.17245/jdapm.2016.16.3.199 PMC5586557

[9] Alshuaibi AF, Aldarwish M, Almulhim AN, Lele GS, Sanikommu S, Raghunath RG (2021) Prevalence of dental fear and anxiety and its triggering factors in the dental office among school-going children in Al Ahsa. Int J Clin Pediatr Dent 14(2):286–292 34413608 10.5005/jp-journals-10005-1925 PMC8343675

[10] Saheer A, Majid SA, Raajendran J, Chithra P, Chandran T, Mathew RA (2022) Effect of dental anxiety on oral health among the first-time dental visitors: a hospital-based study. J Pharm Bioallied Sci 14(Suppl 1):S394–s8 36110809 10.4103/jpbs.jpbs_632_21 PMC9469323

[11] El-Sharkawi HF, El-Housseiny AA, Aly AM (2012) Effectiveness of new distraction technique on pain associated with injection of local anesthesia for children. Pediatr Dent 34(2):e35–e38 22583875

[12] Al-Khotani A, Bello LA, Christidis N (2016) Effects of audiovisual distraction on children’s behaviour during dental treatment: a randomized controlled clinical trial. Acta Odontol Scand 74(6):494–501 27409593 10.1080/00016357.2016.1206211 PMC4960510

[13] AlDhelai TA, Khalil AM, Elhamouly Y, Dowidar KML (2021) Influence of active versus passive parental presence on the behavior of preschoolers with different intelligence levels in the dental operatory: a randomized controlled clinical trial. BMC Oral Health 21(1):420 34454468 10.1186/s12903-021-01781-z PMC8401033

[14] Passos De Luca M, Massignan C, Bolan M, Butini Oliveira L, Aydinoz S, Dick B et al (2021) Does the presence of parents in the dental operatory room influence children’s behaviour, anxiety and fear during their dental treatment? A systematic review. Int J Paediatr Dent 31(3):318–336 33258144 10.1111/ipd.12762

[15] Song ES, Kim WH, Lee BH, Han DW, Lee JH, Kim B (2021) Assessment of color perception and preference with eye-tracking analysis in a dental treatment environment. Int J Environ Res Public Health 18(15):7981 34360272 10.3390/ijerph18157981 PMC8345375

[16] Celine GR, Cho VVY, Kogan A, Anthonappa RP, King NM (2021) Eye-tracking in dentistry: what do children notice in the dental operatory? Clin Oral Investig 25(6):3663–3668 33196871 10.1007/s00784-020-03689-4

[17] Cho VY, Hsiao JH, Chan AB, Ngo HC, King NM, Anthonappa RP (2022) Understanding children’s attention to dental caries through eye-tracking. Caries Res 56(2):129–137 35398845 10.1159/000524458 PMC9254305

[18] Leandro de Oliveira W, Saga AY, Ignácio SA, Rodrigues Justino EJ, Tanaka OM (2019) Comparative study between different groups of esthetic component of the Index of Orthodontic Treatment Need and eye tracking. Am J Orthod Dentofac Orthop 156(1):67–74 10.1016/j.ajodo.2018.07.026 31256841

[19] Bhadila GY, Alyafi DA (2023) The use of eye-tracking technology in pediatric orofacial clefts: a systematic review and meta-analysis. Child (Basel) 10(8):1425 10.3390/children10081425 PMC10453381 37628424

[20] ten Berge M, Veerkamp JS, Hoogstraten J, Prins PJ (2002) Childhood dental fear in the Netherlands: prevalence and normative data. Community Dent Oral Epidemiol 30(2):101–107 12000350 10.1034/j.1600-0528.2002.300203.x

[21] Alamiri B, Alkhamis MA, Naguy A, Alenezi HF, Al Shekaili M (2024) Anxiety disorders among children and adolescents during COVID-19 lockdowns and school closures: a cross-sectional study in Kuwait. Front Psychiatry 15:1322745 38410676 10.3389/fpsyt.2024.1322745 PMC10895000

[22] Alzahrani AAH (2020) Scoping review of dental anxiety among children and adolescents in Saudi Arabia. Interventions Pediatr Dentistry Open Access J 4(1):276–282

[23] Shim YS, Kim AH, Jeon EY, An SY (2015) Dental fear & anxiety and dental pain in children and adolescents; a systemic review. J Dent Anesth Pain Med 15(2):53–61 28879259 10.17245/jdapm.2015.15.2.53 PMC5564099

[24] Oktay EA, Koçak MM, Şahinkesen G, Topçu FT (2009) The role of age, gender, education and experiences on dental anxiety. Age (years) 20:29

[25] Celine G, Cho V, Kogan A, Anthonappa R, King N (2018) Eye-tracking in dentistry: What do children notice in the dentist? J Dent 78:72–75 30114444 10.1016/j.jdent.2018.08.006

[26] Al Tuwirqi AA (2024) Eye-Tracking Technology in Dentistry: A Review of Literature. Cureus 16(2):e55105. 10.7759/cureus.55105 38558726 PMC10978815

[27] Bhadila GY, Alsharif SI, Almarei S, Almashaikhi JA, Bahdila D (2023) Visual analysis of panoramic radiographs among pediatric dental residents using eye-tracking technology: a cross-sectional study. Child (Basel) 10(9):1476 10.3390/children10091476 PMC10527960 37761436

[28] Almashaikhi J, Elkhodary H, Bhadila G, Felemban O, Al Tuwirqi AA (2025) Diagnostic accuracy and radiographic interpretation of pre-eruptive intra-coronal resorption among dental practitioners using eye-tracking technology. Digit Health 11:20552076251315620. 10.1177/20552076251315620 39949845 PMC11822827

[29] Mogg K, Bradley BP (2016) Anxiety and attention to threat: Cognitive mechanisms and treatment with attention bias modification. Behav Res Ther 87:76–108 27616718 10.1016/j.brat.2016.08.001

